# School engagement and student burnout among medical and health science students in Saudi Arabia-cross-sectional study

**DOI:** 10.1038/s41598-025-12879-7

**Published:** 2025-08-03

**Authors:** Amal Mohammed Qasem Surrati, Walaa Abdullah Mumena, Shymaa Abdullah Damfo, Albraa Badr Alolayan, Marwan M. A. Aljohani, Abdulaziz Mofdy Almarwani, Hanaa Mohammed Surrati

**Affiliations:** 1 Family, Community and medical education Department, College of Medicine, Taibah University, Madinah, Saudi Arabia; 2 Clinical Nutrition Department, College of Applied Medical Sciences, Taibah University, Madinah, Saudi Arabia; 3 Pharmacognosy and Pharmaceutical Chemistry Department, College of Pharmacy, Taibah University, Madinah, Saudi Arabia; 4 Department of Oral & Maxillofacial Diagnostic Sciences, Dental College and Hospital, Taibah University, Madinah, Saudi Arabia; 5 Department of Physical Therapy, College of Medical Rehabilitation Sciences, Taibah University, Madinah, Saudi Arabia; 6 Department of Nursing Administration and Education, College of Nursing, Taibah University, Madinah, Saudi Arabia

**Keywords:** School engagement, Student burnout, Medical, Health science, Saudi arabia, Health care, Medical research

## Abstract

School engagement and burnout are contributing factors influencing academic student performance. Student burnout is a serious concern in higher education, where it can result in poor mental and physical health and school dropout. Although recent studies indicate the significance of the relationship between academic engagement and achievement, little is known about how academic engagement and burnout affect medical sciences students in the Madinah region of Saudi Arabia. We aimed to assess the level of school engagement and student burnout among medical and health science students in Madinah, Saudi Arabia. Factors associated with school engagement and student burnout were also explored. A cross-sectional data of 297 students last year medical and health science undergraduate students who were recruited from Taibah University, Madinah. The online survey was shared with students to collect data on sample characteristics, school engagement (using the modified version of the University Student Engagement Inventory), and student burnout (using the modified version of the Maslach Burnout Inventory Student Survey). The data showed that 57.5% of medical science students in the sample were academically engaged, and 55.9% experienced burnout, indicating a moderate to high level of student engagement and burnout. However, there was no correlation between school engagement and student burnout (*r* = 0.27) among the sample in this study. Nevertheless, school engagement was affected by factors such as student living status (*p* = 0.036), dropout thoughts (*p* = 0.008), and cumulative GPA (*p* = 0.001). In addition to dropout thoughts and GPA, the student burnout level was also predicted by the college (*p* = 0.012), program duration (*p* < 0.001), sex (*p* = 0.016), and average sleeping hours per day (*p* = 0.019). These results imply that school engagement and burnout may not be directly related or opposites and can interact in a variety of ways depending on factors including student personality and lifestyle, as well as academic and institutional influences. Many factors were linked to school engagement and student burnout. Interventions that aim to reduce student burnout should be tailored based on many factors including program duration and sex of students.

**Supplementary Information:**

The online version contains supplementary material available at 10.1038/s41598-025-12879-7.

## Introduction

School engagement in higher education refers to the time and energy students devote to a given learning activity; growing evidence identifies engagement as a critical component of the learning process^1^, more clarification what is Learning engagement? Is a key factor tied to academic success is the positive, fulfilling state shown by students during learning, marked by energy, devotion, and concentration^2^.

The “four-dimensional theory” mainly involves academic engagement, including behaviors related to direct participation in the learning process; Social engagement, which includes students’ behavior in observing classroom discipline, lecturer-student interaction, and peer interaction; Cognitive engagement refers to the deep thinking required to understand complex concepts; Affective engagement refers to the sense of identity and belonging to the school^3^. Many medical and health schools have a limited concept of school engagement, typically thinking of it as being involved only in lecture halls, though the concept is much broader. Students should be engaged in the college’s strategic planning process, accreditation, and quality assurance activities^4^.

School engagement has been directly linked to learning outcomes, critical thinking skills, academic achievement, and learning satisfaction^5,6^; the association between school engagement and academic achievement is well established^7–9^. Thus, there is growing interest in engagement as an indicator of the quality of education in medical schools^10^. However, an increasing number of studies are reporting elevated levels of burnout among medical and health science students, which may limit the quality of education provided in medical and health schools^11,12^. A study conducted among medical students enrolled in public universities in Uganda reported a prevalence of burnout of 54.5%^11^. A study conducted in Riyadh, Saudi Arabia among medical students at Alfaisal University reported a lower prevalence of burnout (13.2%)^13^. Engagement was found to be inversely associated with the level of burnout among pharmacy undergraduate students^14^.

Most existing research on student engagement and burnout focuses on the Riyadh region in Saudi Arabia. There is a lack of research targeting medical and health science students in the Madinah region specifically. While Bahlaq and colleagues studied medical students in the Western region, including Madinah, the work focused on burnout in dental students specifically. It cannot be generalized to other medical and health sciences students^36^.

The current research, including the study conducted by Altannir and colleagues, has extensively studied burnout syndrome specifically and overlooked its relation to school engagement. Thus, there is inadequate data in the existing research on the interplay between student engagement and burnout in the same study sample as well as insufficient consideration of the other region^13^.

The government of Saudi Arabia is making significant efforts to improve the quality of health education and healthcare services; however, data concerning engagement and burnout among medical and health science students are still limited. It is crucial to explore the level of school engagement and prevalence of burnout among medical and health science students in public universities in all regions of Saudi Arabia. A better understanding of these factors is important to address issues that may limit the quality of education provided in medical and health science programs in the country. In this study, we aimed to assess the level of school engagement and student burnout among medical and health science students in Madinah, Saudi Arabia. Factors associated with school engagement and student burnout were also explored.

## Methods

### Study design and population

A cross-sectional design was used in this study, which is appropriate for assessing the prevalence of school engagement and student burnout, as well as identifying associated factors at a single point in time. We recruited last year medical and health science undergraduate students of Taibah University, Madinah. Students of six colleges were included (College of Medicine, College of Applied Medical Sciences, College of Medical Rehabilitation Sciences, College of Pharmacy, College of Nursing, College of Dentistry). These students were selected as they are more likely to experience high academic demands and stressors that may contribute to burnout. Inclusion criteria were: being enrolled as a final-year student in one of the six colleges; agree to participate; ability to complete the survey in English. Absent students at the time of data collection were excluded.

The sample size for this study was determined based on the requirements for conducting multiple linear regression analysis, which was used to explore factors associated with school engagement and student burnout. Following Green’s (1991) recommendation for regression models, a minimum of *N* ≥ 50 + 8*m* is required, where *m* is the number of predictors. Assuming 10 predictors (e.g., age, gender, GPA, sleep hours, smoking status, college, screen time, marital status, employment, and income), the minimum sample size would be 130 participants. However, to ensure greater statistical power (90%) and detect a small to moderate effect size (f² = 0.10) at a significance level of α = 0.05, a more robust estimate using G*Power software suggests a minimum of approximately 172 participants. Accounting for potential non-responses or incomplete data, the target sample size was increased by 20%, resulting in a final required sample of 206 participants at minimum^15^.

The study protocol was reviewed and approved by the ethical committee of the Applied Medical Sciences, Taibah University (Certificate number 2024/188/401 NAMS). All procedures followed the ethical standards of research involving human participants.

### Data collection

Data were collected from students via an online survey (Google Form) in March 2024 for two weeks. The link to the survey was shared with students during class which take between 10 and 15 min; the link was also sent to the group leaders to share in the class group to enhance the participation of students. Prior to accessing the questionnaire, all participants were presented with an information sheet outlining the study’s objectives, confidentiality assurances, and their rights as participants. They were required to provide informed consent by selecting an “I agree to participate” option before proceeding with the survey. To prevent multiple entries, Google Form settings were configured to allow one response per email address. The survey, which was in English, gathered information concerning sample characteristics (college, sex, age, marital status, employment status, family monthly income, living status, cumulative GPA, thought about dropping the course, anthropometrics (height and weight to calculate body mass index (BMI)), smoking status, sleeping hours per day, and screen time per day for non-education purposes). Data about school engagement and student burnout were also collected.

### Assessment of school engagement and student burnout

School engagement was assessed using the modified version of the University Student Engagement Inventory (USEI)^14^. This tool collects information concerning the frequency of school engagement using 15 items that are divided into three categories (behavioral engagement, emotional engagement, and cognitive engagement). Response options were a seven-point rating scale ranging from never (score of zero) to always (score of 6). The total score of school engagement was then calculated with a maximum score of 90. The internal consistency of this tool was excellent (Cronbach’s alpha = 0.89).

Student burnout was assessed using the modified version of the Maslach Burnout Inventory Student Survey (MBI-SS)^16^. This tool collects data concerning the frequency and severity of student burnout using 15 items that are divided into three categories (exhaustion, cynicism, and professional efficacy). Response options were a seven-point rating scale ranging from never (score of zero) to always (score of 6). The total score of student burnout was then calculated based on the frequency and severity of burnout with a maximum score of 195. The internal consistency of this tool was excellent (Cronbach’s alpha = 0.82).

### Statistical analysis

Continuous data are described as mean ± standard deviation (SD) and median, whereas categorical variables are described as frequencies and percentages (%). To explore the associations between two categorical variables, Fisher’s exact test was used. The Shapiro-Wilk test was used to assess the normality of the distribution of continuous variables. A total score of school engagement and student burnout were normally distributed. Pearson correlation was used to explore the association between school engagement and student burnout. Independent t-tests and ANOVA were used to compare the mean of different groups. post-hoc Tukey test was used to further explore the significant association reported by the ANOVA test; Bonferroni adjustment was used to correct for multiple testing in post-hoc tests performed. Simple linear regression analyses were conducted to examine the association between various factors (such as college, sex, age group, marital status, etc.) and the two dependent variables: school engagement and student burnout. A total of 14 separate regression models were run for each outcome to assess the individual effect of each independent variable.A significance level of 95% was used in this study. Data were analyzed using SPSS (IBM Corp. Released 2011. IBM SPSS Statistics for Windows, Version 20.0. Armonk, NY: IBM Corp).

## Results

### Sample characteristics

A total of 297 students were included in the final analysis of this study after excluding 62 students (17.2%) who were not in their final year of the academic program. 23% (*n* = 68) of the students were from the Applied Medical Sciences, whereas 10.4% (*n* = 31) were from the College of Dentistry. The proportion of female students included in this study was 53.9% (*n* = 160). Over half of the study sample aged between 21 and 22 years (52.9%, *n* = 157), with the majority of them being single (98.0%, *n* = 291). 97% of students (*n* = 288) were unemployed, with 26.9% of students (*n* = 80) reporting a family income of > SR 20,000 per month. Most of the students (92.9%, *n* = 276) reported living with their family. Over one-third of the students (37.7%, *n* = 112) thought about dropping out of the program, while 53.9% of students (*n* = 160) reported a cumulative GPA between 4.50 and 5.00 (grade A). 54% of students (*n* = 159) were within the healthy weight range (BMI 18.5–24.9 kg/m^2^); 81.8% of students (*n* = 243) never smoked. Over half of the students (54.2%, *n* = 161) reported sleeping < 7 h per day on average, whereas 90.2% of students (*n* = 268) reported media use for non-educational proposes for > 2 hours per day (Table 1).

**Table 1:** Sample characteristics (*n* = 297).

Variable	*n*	%
College		
Applied Medical Sciences (4-year program)	68	22.9
Medical Rehabilitation Sciences (4-year program)	57	19.2
Nursing (4-year program)	50	16.8
Pharmacy (5-year program)	39	13.1
Medicine (6-year program)	52	17.5
Dentistry (6-year program)	31	10.4
Sex		
Male	137	46.1
Female	160	53.9
Age group		
21–22 years	157	52.9
23–24 years	129	43.4
> 24 years	11	3.70
Marital status		
Single	291	98.0
Married	6	2.00
Employment status		
Unemployed	288	97.0
Employed	9	3.00
Family monthly income in Saudi Riyal (SR)		
< SR 6000	53	17.8
SR 6000–10,999	51	17.2
SR 11,000–15,999	67	22.6
SR 16,000–20,999	46	15.5
> SR 21,000	80	26.9
Living status		
Living alone	12	4.00
Living with family	276	92.9
Living in dormitory	8	2.70
Living with friends	1	0.30
Thought about dropping out of the program		
No	185	62.3
Yes	112	37.7
Cumulative GPA		
A (4.50–5.00)	160	53.9
B (3.75–4.49)	113	38.0
C (3.74–2.75)	19	6.40
D (2.74- 2.00)	5	1.70
Weight status		
Underweight (BMI < 18.5 kg/m^2^)	49	16.5
Healthy weight (BMI 18.5–24.9 kg/m^2^)	159	53.5
Overweight (BMI 25.0–29.9 kg/m^2^)	54	18.2
Obesity (BMI ≥ 30.0 kg/m^2^)	35	11.8
Smoking status		
Never smoked	243	81.8
Previously smoked	11	3.70
Current smoker	43	14.5
Sleeping hours per day		
< 7 h	161	54.2
≥ 7 h	136	45.8
Screen time per day		
≤ 2 h	29	9.80
> 2 h	268	90.2

$1 = SR 3.75.

### School engagement and student burnout

Distribution of the responses of the students for the USEI and MBI-SS is provided as **Supplementary Materials**. (**Table ****S1**, **Table S2)** Students who responded to items 3,4,6, and 26 by “Always” were 149 (50.2%), 131 (44.1%), 129 (43.4%), and 85 (28.6%), respectively. Students who responded to item 1 by “Almost always” were 78 (26.3%). Students who responded to item 22 by “Often” were 63 (21.2%). Students who responded to items 5,14,15,16, 17,19,25,28, and 32 by “Sometimes” were 78 (26.3%), 81 (27.3%), 105 (35.4%), 92 (31.0%), 78 (26.3%), 89 (30.0%), 65 (21.9%), and 75 (25.3%), respectively. None of the students responded to any of the items included in the USEI by “Regularly”, “Almost never”, or “Never”.

**Fig. 1:**
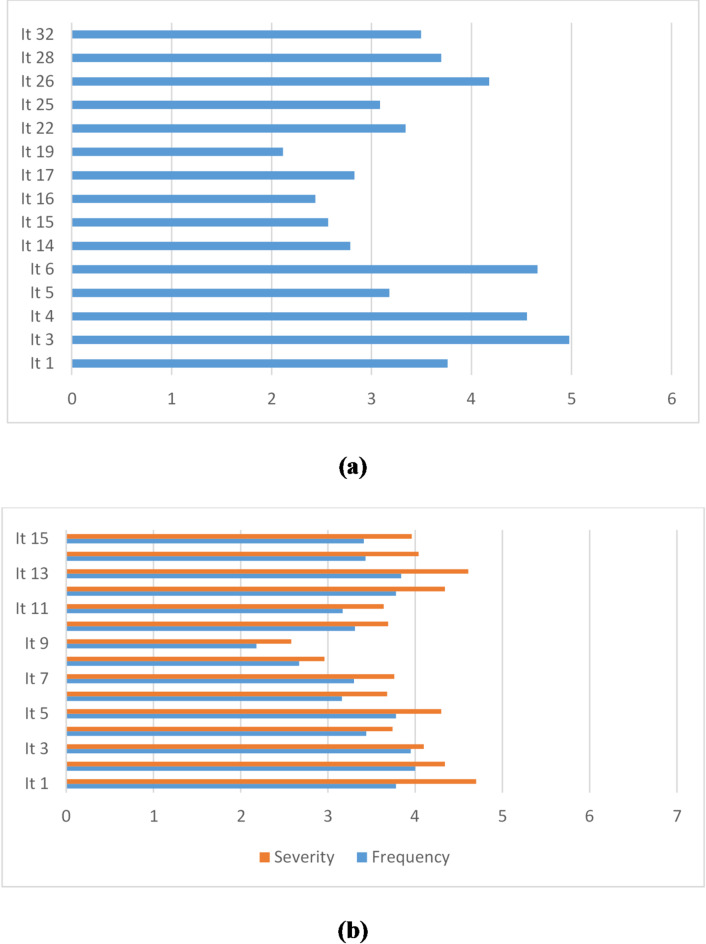
Frequency of school engagement (**a**); frequency and severity of student burnout (**b**).

Students who responded to items 1, 2, 3, 5, 12, 13, and 15 were 63 (21.2%), 73 (24.6%), 79 (26.6%), 70 (23.6%), 75 (25.3%), 87 (29.3%), and 61 (20.5%). Students who responded to item 14 by “Every week” were 67 (22.6%). Students who responded to items 4, 10, and 15 by “A few times a month” were 67 (22.6%), 70 (23.6%), and 61 (20.5%), respectively. Students who responded to items 6, 7, 8, 9, and 11 by “Monthly” were 60 (20.2%), 69 (23.2%), 56 (18.9%), 62 (20.9%), and 69 (23.2%), respectively. Mean school engagement was 51.7 ± 15.4 (score out of 90, minimum 7 and maximum 90)) which shows a limited level of school engagement (57.5%), while mean student burnout was 109 ± 30.5 (score out of 195, minimum 30 and maximum 195) which indicates a high level of student burnout (55.9%). Descriptive data on school engagement and student burnout are provided in Table 2; Fig. 1.

**Fig. 2:**
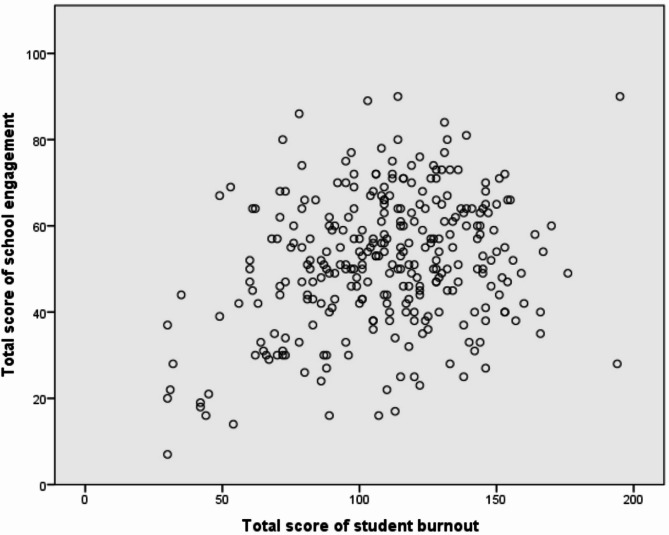
Correlation between school engagement and student burnout.

Although a statistically significant relationship was found between school engagement and student burnout (*r* = 0.27, *p* < 0.001), the strength of this correlation is considered negligible. (Fig. 2).

**Table 2:** Descriptive data on school engagement and student burnout.

Factor	Item	Mean ± SD	Median	Skewness
School engagement				
Behavioral engagement	It 1. Pay attention in class	3.76 ± 1.59	4	− 0.33
	It 3. Follow the school’s rules	4.98 ± 1.37	6	− 1.37
	It 4. Do the homework on time	4.56 ± 1.66	5	− 0.86
	It 5. Ask questions and participate in debates	3.18 ± 1.87	3	0.10
	It 6. Participate actively in group assignments	4.66 ± 1.61	5	− 1.12
Emotional engagement	It 14. Do not feel very accomplished at school	2.79 ± 1.69	2	0.30
	It 15. Feel excited about the schoolwork	2.57 ± 1.67	2	0.41
	It 16. Like being at school	2.44 ± 1.73	2	0.36
	It 17. Interest in the schoolwork	2.83 ± 1.77	3	0.16
	It 19. The classroom is an interesting place	2.11 ± 1.75	2	0.65
Cognitive	It 22. Self-questioning about understanding the readings	3.34 ± 1.61	3	− 0.15
	It 25. Talk to other people on matters that I learned in class	3.08 ± 1.68	3	0.12
	It 26. Try to solve problems when do not understand the meaning of a word	4.18 ± 1.64	4	− 0.56
	It 28. Try to integrate the acquired knowledge in solving new problems	3.70 ± 1.57	4	− 0.11
	It 32. Try to integrate subjects from different disciplines into my general knowledge	3.49 ± 1.58	4	0.02
Student burnout (frequency and severity)				
Exhaustion	It 1. Feel emotionally drained by studies	8.05 ± 3.46	8	− 0.15
	It 2. Feel used up at the end of a studies day	8.33 ± 3.38	8	− 0.26
	It 3. Feel tired when wake up in the morning	8.04 ± 3.55	8	− 0.23
	It 4. Studying or attending a class is really a strain	7.18 ± 3.63	7	0.10
	It 5. Feel burned out from the studies	8.08 ± 3.67	8	− 0.18
Cynicism	It 6. Become less interested in the studies	6.85 ± 3.80	7	0.10
	It 7. Become less enthusiastic about the studies	7.05 ± 3.60	7	0.14
	It 8. Become more cynical about the usefulness of the studies	5.62 ± 4.02	5	0.33
	It 9. Doubt the significance of the studies	4.76 ± 3.78	4	0.54
Professional efficacy	It 10. Can effectively solve the problems of the studies	7.00 ± 3.12	7	0.02
	It 11. Believe in an effective contribution to the classes	6.81 ± 3.73	7	0.06
	It 12. Itself consider a good student	8.12 ± 3.79	8	− 0.26
	It 13. Feel stimulated when achieve study goals	8.44 ± 3.91	9	− 0.37
	It 14. Learn many interesting things in the studies	7.48 ± 3.46	8	− 0.09
	It 15. Feel confident in the class	7.36 ± 3.84	7	0.07

### Factors associated with school engagement and student burnout

Table 3 illustrates the associations between characteristics of students, school engagement, and student burnout. The mean score of school engagement was significantly higher among students who did not think about dropping out of the program compared to students who thought about dropping out of the program (53.5 ± 14.7 vs. 48.6 ± 16.1, respectively, *p* = 0.008). The mean score of school engagement was significantly different across the different cumulative GPA groups (*p* = 0.006). Post-hoc test indicated no significant difference across the GPA groups after correcting for multiple tests.

The mean score of student burnout was significantly different across the different colleges (*p* < 0.001). Post-hoc test indicated that students enrolled in the Nursing program reported significantly lower mean scores of burnout compared to students enrolled in Pharmacy and Dentistry (*p* < 0.001 and *p =* 0.002, respectively). Additionally, data obtained using an independent t-test confirm that students enrolled in a program that is more than 4 years in length have significantly higher scores of burnout compared to students enrolled in a 4-year program (118 ± 29.8 vs. 103 ± 29.7, respectively, *p* < 0.001). Female students reported significantly higher scores of student burnout compared to male students (113 ± 29.2 vs. 105 ± 31.6, respectively, *p* = 0.016). The mean score of student burnout was significantly higher among students who thought about dropping out of the program compared to students who did not think about dropping out of the program (114 ± 35.0 vs. 106 ± 27.1, respectively, *p* = 0.021). The mean score of student burnout was significantly different across the different cumulative GPA groups (*p* < 0.001). Post-hoc tests indicated that students with cumulative GPAs of “A” and “B” reported significantly lower mean scores of burnout compared to students with cumulative GPAs of “D” (*p* = 0.001 and *p =* 0.007, respectively). The mean score of student burnout was significantly higher among students who sleep < 7 h per day on average compared to students who sleep for ≥ 7 h per day (113 ± 29.8 vs. 105 ± 31.0, respectively, *p* = 0.019).

**Table 3:** Associations between characteristics of students, school engagement, and student burnout.

	School engagement Score out of 90	Student burnout Score of 195
College		
Applied Medical Sciences	49.8 ± 15.8	108 ± 27.3
Medical Rehabilitation Sciences	53.3 ± 16.4	106 ± 28.6
Nursing	55.2 ± 17.2	93.4 ± 32.3
Pharmacy	52.3 ± 11.9	124 ± 20.4
Medicine	51.0 ± 13.1	112 ± 28.1
Dentistry	47.4 ± 16.6	120 ± 39.9
*p-value*	0.242	< 0.001*
Program duration		
4 years	52.5 ± 16.4	103 ± 29.7
> 4 years	50.5 ± 13.8	118 ± 29.8
*p-value*	0.281	< 0.001*
Sex		
Male	50.7 ± 15.9	105 ± 31.6
Female	52.5 ± 14.9	113 ± 29.2
*p-value*	0.320	0.016*
Age group		
21–22 years	53.0 ± 15.9	107 ± 27.6
23–24 years	50.1 ± 15.0	112 ± 33.1
> 24 years	49.5 ± 11.9	109 ± 39.9
*p-value*	0.257	0.418
Marital status		
Single	51.7 ± 15.5	109 ± 30.6
Married	48.2 ± 13.5	102 ± 31.1
*p-value*	0.295	0.535
Employment status		
Unemployed	51.8 ± 15.3	110 ± 30.3
Employed	46.7 ± 19.7	89.6 ± 32.3
*p-value*	0.295	0.050
Family monthly income in Saudi Riyal (SR)		
< SR 6000	50.7 ± 16.6	111 ± 29.5
SR 6000–10,999	55.2 ± 14.3	107 ± 29.7
SR 11,000–15,999	49.9 ± 15.8	108 ± 34.3
SR 16,000–20,999	50.0 ± 14.5	108 ± 28.4
> SR 21,000	52.5 ± 15.4	111 ± 30.1
*p-value*	0.345	0.930
Living status		
Living alone	55.2 ± 20.1	97.4 ± 37.4
Living with family	51.8 ± 15.2	110 ± 30.1
Living in dormitory	46.1 ± 10.9	114 ± 26.8
Living with friends	16.0 ± NA	44.0 ± NA
*p-value*	0.070	0.084
Thought about dropping out of the program		
No	53.5 ± 14.7	106 ± 27.1
Yes	48.6 ± 16.1	114 ± 35.0
*p-value*	0.008*	0.021*
Cumulative GPA		
A (4.50–5.00)	53.8 ± 15.1	114 ± 28.8
B (3.75–4.49)	50.2 ± 15.4	105 ± 30.6
C (3.74–2.75)	47.3 ± 12.4	106 ± 32.7
D (2.74- 2.00)	33.8 ± 19.8	61.0 ± 22.1
*p-value*	0.006*	< 0.001*
Weight status		
Underweight (BMI < 18.5 kg/m^2^)	51.2 ± 12.9	114 ± 26.6
Healthy weight (BMI 18.5–24.9 kg/m^2^)	51.9 ± 16.1	110 ± 30.1
Overweight (BMI 25.0–29.9 kg/m^2^)	50.3 ± 15.6	106 ± 34.0
Obesity (BMI ≥ 30.0 kg/m^2^)	53.3 ± 15.4	103 ± 32.1
*p-value*	0.823	0.314
Smoking status		
Never smoked	51.9 ± 15.3	108 ± 28.5
Previously smoked	46.9 ± 16.8	107 ± 36.6
Current smoker	51.7 ± 15.8	114 ± 39.2
*p-value*	0.581	0.509
Sleeping hours per day		
< 7 h	52.2 ± 14.9	113 ± 29.8
≥ 7 h	51.0 ± 16.1	105 ± 31.0
*p-value*	0.419	0.019*
Screen time per day		
≤ 2 h	55.4 ± 14.6	112 ± 33.6
> 2 h	51.3 ± 15.5	109 ± 30.3
*p-value*	0.171	0.624

*Significance at 95% confidence level. Data presented in the table were obtained using independent t-test and ANOVA.

Simple linear regression analysis indicated that school engagement was predicted by student living status (beta (B)= -6.57, Standard error (SE) = 3.13 [95% Confidence Interval (CI): -12.7 to -0.42], *p* = 0.036),The results suggest that students living alone or with family showed higher levels of engagement compared to those living in other arrangements. Thought about dropping out of the program also predicted school engagement significantly (B= -4.90, SE = 1.83 [95% CI: -8.49 to 1.30], *p* = 0.008), with students considering dropping out showing lower engagement levels. Additionally, cumulative GPA (B = 4.27, SE = 1.27 [95% CI: 1.76 to 6.78], *p* = 0.001), indicating that higher GPA scores are associated with increased levels of engagement.

Student burnout was predicted by college (B = 2.63, SE = 1.04 [95% CI: 0.58 to 4.67], *p* = 0.012), indicating differences in burnout levels across colleges. Program duration also significantly predicted student burnout (B = 14.8, SE = 3.51 [95% CI: 7.92 to 21.7], *p* < 0.001), with longer program durations associated with higher levels of burnout. Sex significantly predicted student burnout (B = 8.52, SE = 3.53 [95% CI: 1.58 to 15.5], *p* = 0.016), with female students experiencing higher levels of burnout compared to male students. Thought about dropping out of the program significantly predicted student burnout (B = 8.42, SE = 3.63 [95% CI: 1.28 to 15.6], *p* = 0.021), with students who considered dropping out experiencing higher levels of burnout. Cumulative GPA significantly predicted student burnout (B= -9.44, SE = 2.52 [95% CI: -14.4 to -4.49], *p* < 0.001), with higher GPA associated with lower levels of burnout. Average sleeping hours per day significantly predicted student burnout (B= -8.31, SE = 3.53 [95% CI: -15.3 to -1.36], *p* = 0.019), with students who sleep ≥ 7 h experiencing lower levels of burnout compared to those who sleep < 7 h per day. **(see** Table 4**).**

**Table 4:** Simple linear regression analysis of predictors of school engagement and student burnout.

Variable	Beta	Standard error	*p*-value	95% Confidence Interval	*R*-square
School engagement					
College (Applied Medical Sciences = 1; Medical Rehabilitation Sciences = 2; Nursing = 3; Pharmacy = 4; Medicine = 5; Dentistry = 6)	− 0.32	0.53	0.541	− 1.37 to 0.72	0.00
Program duration (4 years = 1; > 4 years = 2)	− 1.96	1.82	0.281	− 5.54 to 1.61	0.00
Sex (male = 1; female = 2)	1.79	1.79	0.320	− 1.74 to 5.32	0.00
Age group (21–22 years = 1; 23–24 years = 2; > 24 years = 3)	− 2.51	1.57	0.111	− 5.59 to 0.58	0.01
Marital status (single = 0; married = 1)	− 3.56	6.36	0.567	− 16.08 to 8.96	0.00
Employment status (unemployed = 0; employed = 1)	− 5.15	5.22	0.325	− 15.4 to 5.12	0.00
Family monthly income in Saudi Riyal (SR) (< SR 6,000 = 1; SR 6,000–10,999 = 2; SR 11,000–15,999 = 3; SR 16,000–20,999 = 4; > SR 21,000 = 5)	− 0.05	0.62	0.940	− 1.27 to 1.17	0.00
Living status (living alone = 1; living with family = 2; living in dormitory = 3; living with friends = 4)	− 6.57	3.13	0.036*	− 12.7 to − 0.42	0.02
Thought about dropping out of the program (no = 0; yes = 1)	− 4.90	1.83	0.008*	− 8.49 to − 1.30	0.02
Cumulative GPA (A = 4; B = 3; C = 2; D = 1)	4.27	1.27	0.001*	1.76 to 6.78	0.04
Weight status (underweight = 1; healthy weight = 2; overweight = 3; obesity = 4)	0.31	1.03	0.763	− 1.72 to 2.34	0.00
Smoking status (never smoked = 0; previously smoked = 1; Current smoker = 2)	− 0.35	1.25	0.782	− 2.82 to 2.12	0.00
Sleeping hours per day (< 7 h = 1; ≥ 7 h = 2)	− 1.24	1.80	0.491	− 4.77 to 2.30	0.00
Screen time per day (≤ 2 h = 1; > 2 h = 2)	− 2.93	5.98	0.624	− 14.7 to 8.84	0.00
Student burnout					
College (Applied Medical Sciences = 1; Medical Rehabilitation Sciences = 2; Nursing = 3; Pharmacy = 4; Medicine = 5; Dentistry = 6)	2.63	1.04	0.012*	0.58 to 4.67	0.02
Program duration (4 years = 1; > 4 years = 2)	14.8	3.51	< 0.001*	7.92 to 21.7	0.06
Sex (male = 1; female = 2)	8.52	3.53	0.016*	1.58 to 15.5	0.02
Age group (21–22 years = 1; 23–24 years = 2; > 24 years = 3)	3.44	3.11	0.271	− 2.69 to 9.56	0.00
Marital status (single = 0; married = 1)	− 7.84	12.6	0.535	− 32.7 to 17.0	0.00
Employment status (unemployed = 0; employed = 1)	− 20.2	10.3	0.050	− 40.5 to 0.014	0.01
Family monthly income in Saudi Riyal (< SR 6,000 = 1; SR 6,000–10,999 = 2; SR 11,000–15,999 = 3; SR 16,000–20,999 = 4; > SR 21,000 = 5)	0.14	1.23	0.910	− 2.28 to 2.56	0.00
Living status (living alone = 1; living with family = 2; living in dormitory = 3; living with friends = 4)	2.06	6.25	0.742	− 10.2 to 14.4	0.00
Thought about dropping out of the program (no = 0; yes = 1)	8.42	3.63	0.021*	1.28 to 15.6	0.02
Cumulative GPA (A = 4; B = 3; C = 2; D = 1)	9.44	2.52	< 0.001*	4.49 to 14.4	0.05
Weight status (underweight = 1; healthy weight = 2; overweight = 3; obesity = 4)	− 3.84	2.03	0.060	− 7.84 to 0.16	0.01
Smoking status (never smoked = 0; previously smoked = 1; Current smoker = 2)	2.64	2.48	0.289	− 2.25 to 7.53	0.00
Sleeping hours per day (< 7 h = 1; ≥ 7 h = 2)	− 8.31	3.53	0.019*	− 15.3 to − 1.36	0.02
(≤ 2 h = 1; > 2 h = 2)	− 4.13	3.01	0.171	− 10.0 to 1.79	0.01

*Significance at 95% confidence level.

## Discussion

In this study, data show a limited level of school engagement and a high level of student burnout. No correlation was found between school engagement and student burnout. School engagement was predicted by the student’s living status, thoughts about dropping out of the program, and cumulative GPA. Student burnout was predicted by college, program duration, sex, and thoughts about dropping out of the program, whereas cumulative GPA and average sleeping hours per day also predicted student burnout.

The current study showed that student engagement was predicted by students living status, with students living alone having higher engagement levels compared to those living with family, in a dormitory, or with friends. Living with family decreases student engagement by 6.75 units compared to living alone. This reduced engagement may be because students living alone face fewer distractions and have more time to focus on school, leading to higher engagement than students living with family, in a dormitory, or with friends. In addition, living alone might provide a sense of independence and self-reliance which is crucial for personal development during university years. This independence can encourage students to take more responsibility for their academic life, potentially leading to higher engagement^17^. Conversely, students living with their families show higher engagement levels compared to those in a dormitory or with friends. Living with family may provide economic benefits that alleviate financial stress, thereby allowing students to be more engaged in school^18^. Additionally, living with family may provide social and emotional support, creating a stable supportive environment that reduces stress and anxiety related to academic pressure and social life at the university, potentially enhancing academic engagement^19^. On the other hand, previous research has suggested that living with roommates can positively impact student engagement and academic performance, especially for lower-ability students who can benefit from higher-ability roommates^20^. However, this finding may not apply to our study population of medical students, who are considered above average. In this case, living with friends may not improve their school engagement, and having a roommate may harm their engagement level.

In this study student burnout was predicted by program duration. Students enrolled in programs longer than 4 years, such as medicine and dentistry, had significantly higher burnout scores than those in 4-year programs. This is due to the nature of the curriculum and learning outcomes, which require more study time. Studies have shown that students who study around 9 h per day report higher levels of burnout^21^. Programs in medicine, dentistry, and pharmacy typically require over five years of study and students are particularly susceptible to burnout due to academic-related stress factors^22^. There is limited information about the direct association between program duration and student programs in the literature. However, data suggest that students enrolled in medical programs are more likely to experience burnout^23,24^.

Data suggest higher levels of burnout in females compared to male students. Studies conducted among medical and dental students in Saudi Arabia showed that females were more likely to experience burnout compared to male students^13,25,26^. Similar findings have been reported in other settings including Pakistan^27^, Lebanon^28^, the United State^29^, and Morocco^30^. According to Misra and McKean (2000), female students showed significantly increased emotional responses to stressors compared to male students^31^. Additionally, it has been suggested females may be at higher risk of being psychologically vulnerable to the environment of the organization (e.g. gender inequality) compared to their male counterparts^32^.

Burnout is associated with an increased likelihood of serious thoughts of dropping out among medical students, together with a significant negative effect on academic achievement^33,34^. In this study mean score of student burnout was significantly higher among students who thought about dropping out of the program compared to students who did not think about dropping out of the program. There is a negative association between burnout and engagement as both can either be a cause or a consequence of each other^35^. On the other hand, students with cumulative GPAs of “A” and “B” reported a significantly lower mean score of burnout compared to students with cumulative GPAs of “D”. Literature has found that burnout was predicted by low GPA^36^. In another study, they found out that the most likely students to experience burnout were the first-year students as they have to raise their GPA, and a correlation between burnout and students’ GPA showed an improvement in the responder’s GPA^37^.

According to both the National Sleep Foundation and the American Academy of Sleep Medicine, it is recommended that adults obtain 7–9 h of sleep every night^38–40^. Good quality sleep is important for optimal neurocognitive and psychomotor performance as well as physical and mental health^41^. Studies from various countries reported a high prevalence of sleep disturbances among medical students, including sleep deprivation, poor sleep quality, and excessive daytime sleepiness^42^. Poor sleep quality can be caused by a variety of factors, such as stress, long hours of studying, and lack of time management^43,44^. National studies showed that 84% of students sleep less than 8 h each night^43^. More than a third (37%) of the medical students at King Saud University reported abnormal sleep habits^45^, whereas 76% of medical students at King Abdulaziz University reported poor sleep quality^46^. In the United States, 51% of medical students reported poor sleep quality^47^. Poor sleep quality and sleep deprivation are critically linked to higher burnout scores in medical students^48,49^. Studies have found that medical students who experience insufficient sleep are more likely to experience burnout and lack motivation, which can lead to a greater risk of dropout^23^.School engagement plays a critical role in students’ academic and personal development. Research has shown that students who are more engaged tend to achieve higher grades and exhibit enhanced personal and professional skills. A strong psychological connection to the school environment has also been linked to greater learning engagement, improved academic performance and self-efficacy, and reduced academic exhaustion^2^.

Burnout and dropout among medical students have significant personal, psychological, and financial implications. These include emotional distress, health-related issues, and the loss of valuable time, financial resources, and institutional investment. Moreover, burnout is closely linked to increased dropout intentions, which carry both educational and economic repercussions. Student dropout is also regarded as a key indicator of institutional quality in higher education^35^.

This study is the first to explore factors associated with school engagement and student burnout. However, the generalizability of this study might be limited due to the collection of samples from a single university. In addition, the number of students enrolled in some colleges was very limited, which did not allow the study to include an equal proportion of students from each college. As it is a cross-sectional study, causal relationships could not be established. Additionally, the use of convenience sampling may have excluded students with higher burnout levels who chose not to participate. The reliance on a self-administered questionnaire also introduces the potential for recall and social desirability bias.

In conclusion, a limited level of school engagement and a high level of student burnout were observed among the study sample. Several factors were linked to school engagement and student burnout including program duration, sex, academic performance, living status, and sleeping hours. Interventions that aim to reduce student burnout should be tailored based on these factors. Future research should focus on testing approaches that help increase the level of school engagement and limit student burnout to enhance the quality of student’s learning experience and academic performance. Additionally, future research should explore the association between academic program outcomes with school engagement and student burnout.

## Recommendation

Mitigating burnout requires a multifaceted approach grounded in the concept of student engagement and adaptation. Involvement in extracurricular activities—such as community service, music, and physical exercise—combined with emotionally supportive learning environments, fosters resilience. Teaching adaptive skills like problem-solving, emotional expression, and reflective thinking enhances coping capacity. Regular mental health assessments through academic advisory systems are essential to sustaining well-being across the educational trajectory^13^.

As still a critical demand for physicians within the national health system in Saudi Arabia, medical schools should prioritize the implementation of strategies targeting psychological predictors of student well-being. Promoting academic engagement is essential to reducing burnout and mitigating the risk of dropout intentions.

## Supplementary Information

Below is the link to the electronic supplementary material.

Supplementary Material 1

## Data Availability

Data is provided within the manuscript or supplementary information files.
