# Supplementary material for: School engagement and student burnout among medical and health science students in Saudi Arabia-cross-sectional study
Source: Sci Rep. 2025 Aug 3;15:28323. doi: 10.1038/s41598-025-12879-7 (PMC12319107; doi:10.1038/s41598-025-12879-7)
Supplement: Supplementary file 1 — Supplementary Material 1 [file 41598_2025_12879_MOESM1_ESM.docx]

**Supplementary materials**

**Table S1.** Distribution of the responses of the students for the University Student Engagement Inventory (USEI).

| **Items** | **Responses** | | | | | | |
| --- | --- | --- | --- | --- | --- | --- | --- |
| **USEI** | **Never** | **Almost never** | **Sometimes** | **Regularly** | **Often** | **Almost always** | **Always** |
| It 1. Pay attention in class | 7 (2.40) | 14 (4.70) | 61 (20.5) | 41 (13.8) | 54 (18.2) | 78 (26.3) | 42 (14.1) |
| It 3. Follow the school’s rules | 2 (0.70) | 2 (0.70) | 24 (8.10) | 22 (7.40) | 22 (7.40) | 76 (25.6) | 149 (50.2) |
| It 4. Do the homework on time | 3 (1.00) | 13 (4.40) | 34 (11.4) | 30 (10.1) | 34 (11.4) | 52 (17.5) | 131 (44.1) |
| It 5. Ask questions and participate in debates | 19 (6.40) | 40 (13.5) | 78 (26.3) | 26 (8.80) | 49 (16.5) | 36 (12.1) | 49 (16.5) |
| It 6. Participate actively in group assignments | 5 (1.70) | 11 (3.70) | 28 (9.40) | 20 (6.70) | 37 (12.5) | 67 (22.6) | 129 (43.4) |
| It 14. Do not feel very accomplished at school | 24 (8.10) | 44 (14.8) | 81 (27.3) | 45 (15.2) | 56 (18.9) | 19 (6.40) | 28 (9.40) |
| It 15. Feel excited about the school work | 35 (11.8) | 35 (11.8) | 105 (35.4) | 37 (12.5) | 39 (13.1) | 26 (8.80) | 20 (6.70) |
| It 16. Like being at school | 50 (16.8) | 34 (11.4) | 92 (31.0) | 37 (12.5) | 43 (14.5) | 23 (7.70) | 18 (6.10) |
| It 17. Interest in the school work | 37 (12.5) | 26 (8.80) | 78 (26.3) | 54 (18.2) | 43 (14.5) | 30 (10.1) | 29 (9.80) |
| It 19. Classroom is an interesting place | 66 (22.2) | 47 (15.8) | 89 (30.0) | 33 (11.1) | 23 (7.70) | 22 (7.40) | 17 (5.70) |
| It 22. Self questioning about understand the readings | 15 (5.10) | 21 (7.10) | 62 (20.9) | 57 (19.2) | 63 (21.2) | 50 (16.8) | 29 (9.80) |
| It 25. Talk to other people on matters that I learned in class | 16 (5.40) | 32 (10.8) | 89 (30.0) | 33 (11.1) | 56 (18.9) | 43 (14.5) | 28 (9.40) |
| It 26. Try to solve problems when do not understand the meaning of a word | 4 (1.30) | 16 (5.40) | 40 (13.5) | 37 (12.5) | 52 (17.5) | 63 (21.2) | 85 (28.6) |
| It 28. Try to integrate the acquired knowledge in solving new problems | 5 (1.70) | 13 (4.40) | 65 (21.9) | 51 (17.2) | 63 (21.2) | 50 (16.8) | 50 (16.8) |
| It 32. Try to integrate subjects from different disciplines into my general knowledge | 4 (1.30) | 22 (7.40) | 75 (25.3) | 47 (15.8) | 59 (19.9) | 51 (17.2) | 39 (13.1) |

Data presented table are frequencies and percentages.

**Table S2.** Distribution of the responses of the students for the Maslach Burnout Inventory (MBI-SS).

| **Items** | **Responses** | | | | | | |
| --- | --- | --- | --- | --- | --- | --- | --- |
| **MBI-SS** | **Never** | **A few times a year** | **Monthly** | **A few times a month** | **Every week** | **A few times a week** | **Every day** |
| It 1. Feel emotionally drained by studies | 6 (2.00) | 28 (9.40) | 47 (15.8) | 50 (16.8) | 43 (14.5) | 60 (20.2) | 63 (21.2) |
| It 2. Feel used up at the end of a studies day | 5 (1.70) | 19 (6.40) | 42 (14.1) | 51 (17.2) | 44 (14.8) | 63 (21.2) | 73 (24.6) |
| It 3. Feel tired when wake up in the morning | 7 (2.40) | 24 (8.10) | 37 (12.5) | 60 (20.2) | 30 (10.1) | 60 (20.2) | 79 (26.6) |
| It 4. Studying or attending a class is really a strain | 14 (4.70) | 25 (8.40) | 59 (19.9) | 67 (22.6) | 35 (11.8) | 44 (14.8) | 53 (17.8) |
| It 5. Feel burned out from the studies | 6 (2.00) | 26 (8.80) | 50 (16.8) | 54 (18.2) | 40 (13.5) | 51 (17.2) | 70 (23.6) |
| It 6. Become less interested in the studies | 17 (5.70) | 44 (14.8) | 60 (20.2) | 59 (19.9) | 30 (10.1) | 43 (14.5) | 44 (14.8) |
| It 7. Become less enthusiastic about the studies | 9 (3.00) | 40 (13.5) | 69 (23.2) | 52 (17.5) | 34 (11.4) | 49 (16.5) | 44 (14.8) |
| It 8. Become more cynical about the usefulness of the studies | 45 (15.2) | 49 (16.5) | 56 (18.9) | 52 (17.5) | 32 (10.8) | 31 (10.4) | 32 (10.8) |
| It 9. Doubt the significance of the studies | 62 (10.9) | 59 (19.9) | 62 (20.9) | 51 (17.2) | 20 (6.70) | 26 (8.80) | 17 (5.70) |
| It 10. Can effectively solve the problems of the studies | 11 (3.70) | 21 (7.10) | 66 (22.2) | 70 (23.6) | 49 (16.5) | 55 (18.5) | 25 (8.40) |
| It 11. Believe in an effective contribution to the classes | 27 (9.10) | 26 (8.80) | 69 (23.2) | 52 (17.5) | 36 (12.1) | 45 (15.2) | 42 (14.1) |
| It 12. Itself consider a good student | 13 (4.40) | 21 (7.10) | 50 (16.8) | 51 (17.2) | 36 (12.1) | 51 (17.2) | 75 (25.3) |
| It 13. Feel stimulated when achieve study goals | 11 (3.70) | 23 (7.70) | 57 (19.2) | 40 (13.5) | 35 (11.8) | 44 (14.8) | 87 (29.3) |
| It 14. Learn many interesting things in the studies | 8 (2.70) | 29 (9.80) | 60 (20.2) | 51 (17.2) | 67 (22.6) | 42 (14.1) | 40 (13.5) |
| It 15. Feel confident in the class | 15 (5.10) | 32 (10.8) | 60 (20.2) | 61 (20.5) | 29 (9.80) | 39 (13.1) | 61 (20.5) |

Data presented table are frequencies and percentages.
